# Anticancer Potential of Furanocoumarins: Mechanistic and Therapeutic Aspects

**DOI:** 10.3390/ijms21165622

**Published:** 2020-08-06

**Authors:** Salman Ahmed, Haroon Khan, Michael Aschner, Hamed Mirzae, Esra Küpeli Akkol, Raffaele Capasso

**Affiliations:** 1 Department of Pharmacognosy, Faculty of Pharmacy and Pharmaceutical Sciences, University of Karachi, Karachi 75270, Pakistan; salmanahmed@uok.edu.pk; 2 Department of Pharmacy, Abdul Wali Khan University, Mardan 23200, Pakistan; haroonkhan@awkum.edu.pk; 3 Department of Molecular Pharmacology, Albert Einstein College of Medicine, Bronx, NY 10463, USA; michael.aschner@einsteinmed.org; 4 Research Center for Biochemistry and Nutrition in Metabolic Diseases, Institute for Basic Sciences, Kashan University of Medical Sciences, Kashan 8715973474, Iran; mirzaeih911h@mums.ac.ir; 5 Department of Pharmacognosy, Faculty of Pharmacy, Gazi University, Etiler, 06330 Ankara, Turkey; esrak@gazi.edu.tr; 6 Department of Agricultural Sciences, University of Naples Federico II, Via Università 100, 80055 Portici, Italy

**Keywords:** furanocoumarin, apoptosis, autophagy, metastasis, cell cycle arrest

## Abstract

Cancer is one of the most extreme medical conditions in both developing and developed countries around the world, causing millions of deaths each year. Chemotherapy and/or radiotherapy are key for treatment approaches, but both have numerous adverse health effects. Furthermore, the resistance of cancerous cells to anticancer medication leads to treatment failure. The rising burden of cancer overall requires novel efficacious treatment modalities. Natural medications offer feasible alternative options against malignancy in contrast to western medication. Furanocoumarins’ defensive and restorative impacts have been observed in leukemia, glioma, breast, lung, renal, liver, colon, cervical, ovarian, and prostate malignancies. Experimental findings have shown that furanocoumarins activate multiple signaling pathways, leading to apoptosis, autophagy, antioxidant, antimetastatic, and cell cycle arrest in malignant cells. Additionally, furanocoumarins have been shown to have chemo preventive and chemotherapeutic synergistic potential when used in combination with other anticancer drugs. Here, we address different pathways which are activated by furanocoumarins and their therapeutic efficacy in various tumors. Ideally, this review will trigger interest in furanocoumarins and their potential efficacy and safety as a cancer lessening agents.

## 1. Introduction

Cancer exacts one of the greatest medical tolls on humankind, requiring a proactive procedure for prevention and treatment. An enormous number of patients succumb to cancer every year. It is one of the chief reasons for mortality around the world, and the number of cases is continually expanding and estimated to reach 21 million by 2030. The lack of efficient anticancer treatments remains a clinical problem [1,2]. Chemotherapy and/or radiotherapy are the main clinical approaches to cancer treatment, yet both have documented adverse effects [3,4,5,6]. Cancer treatment affects not only rapidly multiplying cancerous cells but also normal body cells (bone marrow, gastrointestinal tract (GIT), and hair follicles); therefore, these treatments may give rise to severe adverse symptoms. Moreover, quick disposal and widespread distribution of the medications in cancer-free organs requires high dosing, which may lead to incremental adverse reactions. Resistance towards malignant growth is another restriction.

Restorative plants have been utilized previously. Phytopharmaceuticals primarily target malignant growth, and hence, they are the most appropriate contender for anticancer medications [1,2]. Nowadays, significant efforts have improved the efficacy of natural anticancer drugs with the appearance of encouraging strategies [7,8].

Furanocoumarins are phytochemicals that have been utilized for quite a while. The *Atharva-Veda*, the Indian hallowed book, portrays the *Psoralea corylifolia* poultice, and the old Egyptians utilized *Ammi majus* for leukoderma (vitiligo). In 1838, 5-Methoxypsoralen (5-MOP) was the first furanocoumarin isolated from *Citrus bergamia* oil by Kalbrunner [9]. Furocoumarins are formed by coumarin and a furan ring combination, resulting in angular or linear isomers depending on the furan ring position. Angelicin and psoralen are basic furocoumarins that act as precursors for angular and linear furocoumarins, respectively. These compounds are, for the most part, biosynthesized by phenylpropanoid and the mevalonic pathways. Furocoumarins are produced in plants of Apiaceae and Rutaceae as well as in Asteraceae, Caryophyllaccae, Fabaceae, Moraceae, and Salvadoraceae for defense against insects, bacterial and fungal predators. They provide antimicrobial and insecticidal activity and behave as natural pesticides [10]. Furocoumarins have promising therapeutic prospects, such as analgesic, anticonvulsive, anticoagulant, hypotensive, antidepressants, antibacterial, antifungal, antiviral, anti-inflammatory, antiallergic [11,12], antioxidants [13], and inhibitors of human carbonic anhydrase isozymes [14], against skin diseases [15,16], hyperproliferative disorders [17,18] and as an anticancer [19]. This review is aimed at evaluating the literature on the anticancer potential of various furanocoumarins through different underlying mechanisms and thereof therapeutic/clinical status.

## 2. Chemistry of Furocoumarins

The exact molecular mechanism of such an activity relies upon the chemical structure of furanocoumarins, which depends on the furan ring and coumarin backbone combination in an angular or linear structure just as the type, location, and the number of the substituents attached [11]. The CH3 presence at C5 improves the tumor properties of psoralen and 5-MOP, paying little heed to the substituent location. The substitution of the methoxy group with an isopentenyloxy moiety in the C5 position prompted abatement in the pro-apoptotic properties of the compound [20,21,22]. Angelicin is the most straightforward angular furanocoumarin and it displays counter cancer properties. Analogous to linear furocoumarins, angular analogs can be substituted with a methoxy or isopentenyloxy group. Methoxy subordinates of angelicin incorporate isobergapten and sphondin. Isobergapten, for example, 5-methoxyangelicine, is a linear isomer of bergapten with a methoxy group joined to the fifth (C5) carbon atom. Thus, sphondin (6-methoxyangelicin) can be considered as an angular analogue of xanthotoxin. The thing that matters is, be that as it may, that the methoxy group is appended to the C6 position in 6-methoxyangelicin and to the C8 atom in the 8-MOP [11].

**Figure 1:**
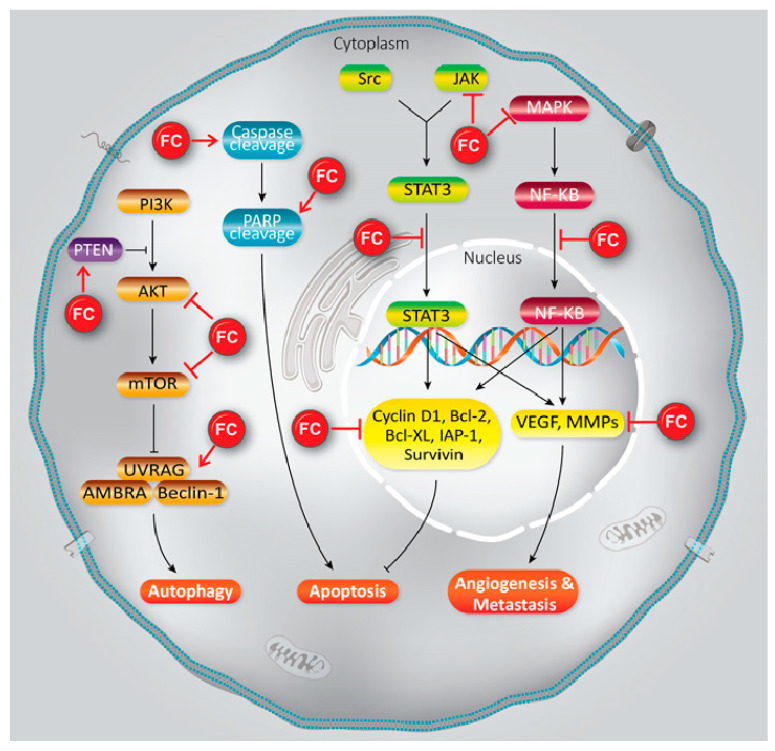
A schema of different molecular mechanisms that are targeted by furanocoumarin. It shows several molecular singling pathways modulation that leads to autophagy, apoptosis, angiogenesis, and metastasis. Black lines: induce, and red lines: inhibit.

Furanocoumarins’ defensive and restorative properties have been observed in leukemia, glioma, breast, lung, renal, liver, colon, cervical, ovarian, and prostate malignancies. Apoptosis, autophagy, antioxidant, cell cycle capture, Nuclear Factor Kappa-light-chain-enhancer of activated B cells (NF-κB) inactivation, Phosphatidylinositol 3-kinase/RAC-α Serine/Threonine-Protein Kinase (PI3K/Akt) inhibition, and p53 modulation incorporate mechanistic insight (Table 1; Figure 1). In this article, we have reviewed the experimental data showed the role of furanocoumarins for cancer prevention and treatment.

## 3. Bioavailability of Furocoumarins

Furanocoumarins are rapidly absorbed from food into the human bloodstream and detected in plasma within 2–15 min after administration [20], and distributed to the skin, blood, liver, brain, spleen, kidney, and testis. In plasma, furanocoumarins bind to albumins and other plasma proteins. Furanocoumarins are metabolized to psoralen and isopsoralen by intestinal bacteria in the digestive tract. Then, furanocoumarins are excreted into urine as hydroxylated or glucuronated products within 1 h after ingestion. They remain in urine as long as 24 h post-administration. It was also observed that furanocoumarins are converted to bergaptol before excretion [11,21,22].

A significant advance in the investigation of the anticancer properties of furanocoumarins was the revelation of their antiproliferative activity arresting cell-cycle capture and causing cell death [23,24,25]. At the cellular levels, furanocoumarins appear to affect actin filaments, which might be valuable in metastasis prevention [26].

## 4. Mechanistic Insights

### 4.1. NF-κB Inactivation

NF-κB and STAT3 inactivation or inhibition cause apoptosis and consequent ineffectiveness of anticancer treatments [23,24]. NF-κB and signal transducer and activator of transcription 3 (STAT3) association triggers inflammation and cancer [25]. Bergamottin enhances tumor necrosis factor (TNF) induced apoptosis in U87 and U251 via NF-κB and STAT3 inhibition [26]. The blocking of NF-κB activity by psoralen (80 μg/mL) leads to inhibition of FADD like IL-1β converting enzyme inhibitory protein (c-FLIP) and inhibitor of apoptosis proteins (IAP), activation of Bax, JNKs, and blocking G1/S phase in KBv200 and K562 [27].

MMP-2,9 decrement, E-cadherin increment, EMT inhibition, and JNK cascade activation are associated with NF-κB inactivation and result in antimetastatic behavior [28,29,30,31,32]. Angelicin inhibits A549 non-small-cell lung carcinoma (NSCLC) growth and metastasis by reducing MMP-2,9; increasing E-cadherin expression levels and JNK, and ERK activation [33]. Bergamottin inhibited the migration abilities of A549, H1299 [34]; HT-1080 cells [35] and human fibrosarcoma HT-1080 cells [36], by decreasing the phorbol 12-myristate 13-acetate (PMA)-initiated enactment of matrix metallo proteinase (MMP)-2,9; c-jun N-terminal kinase (JNK) phosphorylation and EMT inhibition through NF-κB inactivation. Psoralen give similar response in MCF-7/ADR cells [37]. Rac1 blocks drug-induced apoptosis by maintaining Bad in the phosphorylated state [38]. Inactivation of Rac 1 is also responsible for NF-κB inactivation [39]. Bergamottin exhibits antimetastatic behavior in U87 and U251 human glioma cells through Rac1 inactivation and MMP-9 downregulation [26].

In chronic inflammation, immune cells generate excessive ROS and RNS in the inflamed tissue and reinforce the NF-κB mediated inflammatory responses that can lead to tumorigenesis. Therefore, NF-κB down regulation is helpful in cancer prevention and treatment by exerting anti-inflammatory effect [40,41]. NF-κB expresses cytokines, chemokines, and also maintains the inflammatory response through persistent leukocyte activation [42,43]. Sphondin inhibits IL-1β-initiated-COX-2 by NF-κB inactivation in the A549 cell line [44]. Imperatorin reduced IL-6, -1β, and TNF-α discharge and inhibited iNOS and COX-2 by repressing NF-κB in RAW 264.7 (murine-macrophages-cell lines) [45,46]. Bergamottin, Bergapten and Psoralen inhibit ROS/NO generation for anti-inflammatory activity [35,47,48].

### 4.2. PI3K/Akt Inhibition

Activated PI3K/Akt contributes to (i) protection of cells from apoptosis, by inactivation of Bax, Bad, Bak, Bid, MDM2, caspase-9, and Bax and causes activation of cyclic AMP responsive element binding protein (CREB) to induce transcription of Bcl-2, (ii) regulation of cell metabolism; (iii) fatty acid synthesis. In apoptosis, cytosol Bax releases caspase-activating Cyt-c. The caspase-8 (extrinsic apoptosis) or -9 (intrinsic apoptosis), is activated which further activates caspase-3, essential to propagates apoptotic signal [49,50,51,52,53]. Cell shrinkage and chromatin condensation helps in apoptosis [54]. Activated caspase-3 stimulates inhibitor of caspase activated DNAse (ICAD) to release caspase-activated DNAse (CAD), which condenses the chromatin [55]. Angelicin in PC-3 cells [56] and bergamottin in A549 (NSCLC) cell lines [57] cause chromatin condensation. Imperatorin induced selective antitumor effects in SGC-7901 cells without causing too much cytotoxicity to the normal mouse fibroblast cells (3T3 cells). It also induced apoptosis, G1 phase arrest, DNA fragmentation, and downregulation of PI3K/Akt/m-TOR signaling pathway [58]. Methoxsalen (8-MOP) activated caspase-8 -9, Bax/Bcl-2 ratio upregulation, cytochrome-c (Cyt-c) release, and CREB phosphorylation decrement to down-regulates Bcl-2 for apoptosis in SW620 and SK-N-AS cells, through PI3K/Akt down-regulation [51]. Angelicin increases Bax, caspase-3, -9, decreases Bcl-2, Bcl-xL, Mcl-1 in A549 (NSCLC) cells [33], and SH-SY5Y cells [59] to induce intrinsic mitochondria-mediated apoptosis. Angelicin, and in combination, activated caspase-3 and decreased FADD like IL-1β converting enzyme inhibitory protein (c-FLIP) in Caki (renal carcinoma) cells to induce apoptosis [60]. Bergamottin induces apoptosis in human colon carcinoma cell line (RKO) and HT-29 cells by increasing caspase-3, 8, and 9; and PARP cleavage [61]. Bergapten inhibits breast cancer cell line (MCF-7) growth [62] and CRC cell viability [63] via inhibition of Akt. Bergaptol inducesMCF-7 cytotoxicity by the same mechanism [64]. Methoxsalen (8-MOP) inducesHepG2 cells apoptosis by Bax/Bcl-2 increment, MMPs decrement, and Cyt-c release and AIF transposition induction [65]. Imperatorinin duce’s both intrinsic and extrinsic apoptotic pathways by Bcl-2 protein expression downregulation, caspase-3, -7, -8, -9 activation, releases mitochondrial Cyt-c to the cytosol and cleaves poly (ADP-ribose) polymerase (PARP) in HT-29, HL-60 [52,66], SNU 449, HCT-15 [67], and HepG2 cells [68]. Isoimperatorin induces the apoptosis in SGC-7901 and in vivo xenograft model by Bax, caspase-3, -9 increment, and Survivin and Bcl-2 decrement in nude mice [69]. Marmesin inhibits colony formation and induces apoptosis in U937 cells by triggering Bax upregulation, Bcl-2 downregulation, and Bax/Bcl-2 ratio increment [70]. Byakangelicol, cnidicin, imperatorin, isoimperatorin, oxypeucedanin and (+)-oxypeucedanin hydrate from *Angelica dahurica* roots exert cytotoxicity in A549, HCT-15, SK-OV-3, SK-MEL-2, XF498 cells by similar mechanisms [71]. 5-geranyloxy-7-methoxycoumarin from lime suppressesSW480 cell proliferation by apoptosis through caspase-3, -8 activation, regulation of Bcl2, and p38 MAPK phosphorylation inhibition [72]. Psoralen causes Bax increment and attenuates Bcl-2 expression in SMMC7721 human hepatoma cells [73]. Liver X receptor (LXR) regulates lipid metabolism, inflammation and induces apoptosis through caspase-3 activation [74]. LXRs activation increases ABCA1 cholesterol transporters and inducible degrader of the low-density lipoprotein receptor (IDOL) in turn triggering low-density lipoprotein receptor (LDLR) degradation and reducing intracellular cholesterol, thereby reducing SREBPs, tumor growth, and survival. Highly expressed SREBPs play important roles in malignancies, connecting oncogenic signaling to lipid metabolism alterations, leading to rapid tumor growth. PI3K-Akt pathway is inhibited by LXRs [53,75]. Bergapten inhibits liver carcinogenesis (HepG2) by activating LXRs, inhibiting PI3K/Akt that reduced SREBP-1, fatty acid synthase (FASN), stearoyl-CoA desaturase-1 (SCD1), thereby preventing fatty acid synthesis and tumor growth [53].

The antitumor effects of angelicin involves decreased expression of p-VEGFR2 and PI3K/Akt signaling inhibition in HepG2 and Huh-7 cells. In the same study, angelicin was shown to decrease p-VEGFR in mouse liver orthotopic xenograft model [17]. Bergamottin suppresses TGF-β initiated EMT and the cell invasive potential by PI3K, Akt, and mTOR kinases [35].

### 4.3. p53 Modulation

The *p53* controls cell cycle progression and regulates apoptosis and autophagy [76]. *p53* activation promotes *p21* and *p27* expression, which restrain cyclin E and Cdk2 activity, thereby cause cell cycle phase arrest [77]. Bergapten increases *p53* activity that induces *p21* transcription, which, in turn, inhibits G2/M phase in HepG2 cells [11]; triggers G1 arrest in A549, NCI H460 [78] and MCF-7 cells [62]. p53 protein intervenes in two major apoptotic pathways: an “extrinsic pathway” induced by death receptors (TNF proteins such as DR4 and DR5) and an “intrinsic pathway” that regulates Family Bcl-2 proteins [5]. Bergapten (30 and 50 μM) decreased CRC (colorectal cancer) cells’ viability by apoptosis via upregulation of *p53*, *p21*, and PTEN [63]. Furanocoumarin A from Fructus liquidambaris induces the apoptosis in A549 by increasing *p53* [79]. Imperatorin significantly upregulates *p53* and *p21*, which subsequently results in Mcl-1 down-regulation and Bax up-regulation in H23 human lung cancer cell [80] and HT 29 colon cancer cells [52] to inhibit their growth. Psoralen upregulates *p21*, waf and *p53* in cells SKBR-3 MCF-7 cells, causing apoptosis [81,82]. Similarly, 5-geranyloxy-7-methoxy coumarin induces apoptosis in SW480 cells through the activation of tumor suppressor gene *p53* [72].

Psoralen inhibits SMMC7721 cells (human hepatoma cell line) proliferation by ER stress induction by apoptosis. During ER-stress, glucose regulatory protein 78 (GRP78), GRP94, protein kinase R-like ER kinase (PERK), inositol requiring enzyme 1 (IRE1), and activating transcription factor-6 (ATF6), growth arrest and DNA damage inducible protein 34 (GADD34), and ATF4 increment can promote C/E B P homologous protein (CHOP) and high expression of CHOP, thereby inhibiting Bcl-2 and promoting apoptosis [73].

Psoralen causes exosomes formation and secretion reduction through *p53* and PPAR activation in MCF-7/ADR cells, which showed its role against chemotherapy resistance in breast cancer [83].

The antioxidant functions of p53 protein also induces apoptosis and autophagy [5,84]. Imperatorin increases *p53*, in turn, exerting antioxidant effects that may contribute to its anticancer effects [85,86].

### 4.4. Cell Cycle Arrest

Cell cycle progression or arrest is related to cyclin-A, -B1, -D1, -E1 and cyclin B1, cyclin E1, Cdc2, Cdk2 and Wnt/β-catenin [87,88,89]. Angelicin suppresses proliferation of A549 cells; HeLa and SiHa cells by promoting G2/M and G1/G0 phase arrest respectively by cyclin-B1, -E1 and Cdc2 downregulation [33,90].Bergamottin arrests HT-29, RKO and A549 (NSCLC) cells at G2/M by cyclin-A, -B1 and Cdc2 depletion [57,61]. Bergapten (5-MOP) blocks G2/M phase in Hep-G2 with the inhibition of Cdk1 [91]. Bergapten (30 and 50 μM) instigated the G0/G1 and sub-G1 stage capture in CRC cells by a decrease of cyclin E/Cdk2 [63]. Bergaptol arrests G1-phase in MCF-7 [64]; Imperatorin in HT 29 and SGC-7901 cells [52,58] and isoimperatorin in DU145 and SGC-790 cells by increasing Cip1/p21 and Kip1/p27 expression and disrupting Cdk4 kinase synthesis [69,92]. Marmesin captures G2/M and cell migration in U937 [70]. Psoralen arrests at G1 phase in SMMC7721 by Cyclin-E1 reduction [73]. Psoralen arrests G0/G1 by deregulation of Wnt/β-catenin in MCF-7 [93].

### 4.5. Autophagy

Increase in autophagy results in cell growth inhibition and apoptosis [94,95]. Angelicin accumulated microtubule-associated LC3B and upregulated Atg-3,7 and 12-5 in HeLa and SiHa cells [90]. PTEN is downregulated to corneal tumors [96]. Bergapten exhibited autophagy in MCF-7 and ZR-75 by PTEN, AMBRA, Beclin1, PI3KIII and, UVRAG up-regulation and LC3-II conversion [97]. Feroniellin-A from *Feroniella lucida* roots initiates autophagy in A549 by LC3-II conversion; Beclin-1 and Atg-5 enhanced expression [98].

### 4.6. Antioxidant

Oxidative stress is caused by ROS accumulation and high ROS concentration is demanded by cancerous cells. Anticancer phytochemicals with antioxidants properties reduce developing cancer risks [45,99]. Bergamottin, bergaptol, methoxsalen [100], bergapten [101], imperatorin [102], oxypeucedanin [103] and psoralen [104] have been reported to possess antioxidant activities. N-nitrosodiethylamine (NDEA) causes oxidative stress and responsible for the carcinogenic effect. Bergapten has shown to possess anticancer potential in vivo against NDEA-induced liver cancer via antioxidant effects [53]. Hypoxia is a noteworthy component of tumor malignant growth, and it activates hypoxia inducible factor (HIF), thus increasing tumor survival and proliferation [85]. Antioxidants inhibit HIF-1 α activity by scavenging free radicals [105]. Imperatorin has been shown to inhibitHIF-1 in HCT116 due to its antioxidant behavior and mTOR/p70S6K/4E-BP1 and MAPK downregulation [86].

## 5. Role in MDR Cancers

Cancer cell defiance to chemotherapy is one of the significant deterrents for counter cancer medications due to the involvement of different mechanisms. The main causes are related to the increment of multidrug efflux pumps, including P-gp by tumor cells, and showed MDR. Coumarins have a significant role in MDR inversion [106,107,108].

Psoralen inverts the P-gp-instigated MDR in MCF-7/ADR by repressing the efflux capacity of P gp [108] and by hindrance of P-gp ATPase activity [37]. Similarly, isopimpinellin (IC50 value 26 µM) and Phellopterin (IC50 value 8 µM) exhibited cytotoxic activity against MDR HL-60/MX2 (human promyelocytic leukemia cells) and CEM/C1 (human lymphoblastic leukemia cells) respectively [109]. Feroniellin A reduces MDR in A549 cell lines by decreasing P-gp [98]. MDR1rapidly pumps out anticancer drugs, decreases intracellular drug concentrations and leads to the failure of anticancer therapeutics [110]. Similarly, BCRP and MRP overexpression participates in MDR [111,112]. Bergapten and methoxsalen showed cytotoxicity in MDR1, MRP2 BCRP overexpressing gastric (EPG85.257RDB), ovarian (A2780RCIS) and breast (MCF7MX) cancer cell lines which showed reticence of MDR1, BCRP, and MRP efflux functions [113].

ETRα positive and estrogen dependence are seen in 70% of breast cancers. ETRα depletion during initial stages of breast cancer is a potent anticancer approach [114]. Xanthotoxol, bergapten, angelicin, psoralen and isoimperatorin antagonized ETRα activity in MCF-7 cells with IC50 values of 0.72, 1.18, 11.02, 24.08 and 54.32 μM, respectively [115].

Exosomes, secreted from tumor cells to promote tumor progression, such as metastasis and MDR, activate sequestration of anticancer drugs by MDR-1 and P-gp [116]. Psoralen significantly reduces the number of exosomes, which correlates with increased MCF-7/ADR cells’ sensitivity for apoptosis under the influence of chemotherapy. Similar observations were seen in A 549/D16 lung cancer cell lines [11,83,117].

## 6. Furanocoumarins as Adjuvant with Other Anticancer Agents

**Figure 2:**
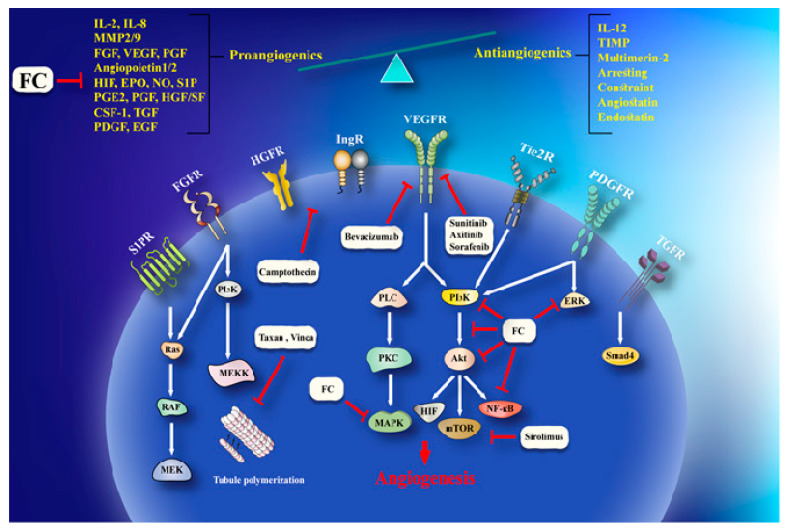
A schema of anti-angiogenesis effects of furanocoumarin. FC via activation/inhibition of a sequences of cellular and molecular pathways exerts its anti-angiogenesis effects. Black lines: induce, and red lines: inhibit.

Invasion and angiogenesis are important targets of FC (Figure 2). In a study, given that bergamottin (50 μM)-potentiated simvastatin (SV) inhibits TNF-induced NF-κB activation and IκBα deterioration, cell-cycle capture in S-phase by boost of p21 and p27 in KBM-5 cells. This impact of co-treatment is associated with decrease incyclin D1 (cell proliferation), Bcl-2, cIAP-1, Bcl-xL and survivin (cell survival), MMP-9 (invasion), and VEGF (angiogenesis) which are directed by NF-κB. CYP P450 34A inhibition by bergamottin enhances SV bioavailability in KBM-5 to synergize SV effects [35,118].Cytochrome P450 upregulation in cancer makes it one of the effective strategy against it. CYP P450 inhibition by furanocoumarins and other phytoconstituents leads to anticarcinogenic effects, down regulating MDR and prolonging the t1/2 of other anticancer drugs [119,120]. Bergamottin, DHBG, bergapten, and bergaptol inhibit CYP P450 34A to potentiate vinblastine effects [121]. Bergamottin, imperatorin, and isopimpinellin repressed CYP P450-1A1, -1A2, -3A4, and -1B1 (involve in carcinogen metabolism), in the MCF-7 cell lines [122]. MDR has been linked to BCRP, MRP, and MDR1 increase that expel cisplatin, daunorubicin, and mitoxantrone are very common in cancer therapeutics [113]. Bergapten and methoxsalen are capable of averting mitoxantrone, cisplatin, and daunorubicin binding to MDR1, BCRP, and MRP and, therefore, impeding their cellular efflux [113]. Bergamottin, DHBG, bergapten, and bergaptol from grapefruit juice inhibit P-gp and MRP2 mediated vinblastine efflux [121]. Long-term use of tamoxifen in breast cancer leads to MDR and an increased risk of endometrial carcinogenesis [123]. Bergapten degrades and depletes ETRα from MCF-7 tamoxifen-resistant cells by inducing SMAD4 protein to block mitogenic signals [62]. The anticancer effects of doxorubicin in HeLa cells are enhanced by Imperatorin via Mcl-1 down-regulation [124]. Imperatorin (100 μM) effectively increasing autophagy in HeLa cells in response to cisplatin (2 μM) by microtubule-associated protein 1A/1B-light chain 3 (LC3) cleavage and the inhibition of Hsp27 and Hsp72 [125]. Imperatorin potentiates the cytotoxicity of cisplatin in hepatocellular carcinoma (HCC) cells (HepG2, HepG3B, PLC, Huh7) by downregulating Mcl-1 expression [126].

## 7. Furanocoumarins and Cancer Risks

PUVA therapy (photoactivated psoralens) is beneficial for patients with vitiligo, psoriasis, and skin diseases. PUVA activates caspase-3, -8 and -9 in Jurkat (human tumor T-cell line) [127]. PUVA, the photoactivated psoralens (5-MOP and 8-MOP) induce apoptosis in MCF-7, ZR-75 and SKBR-3 by caspase and p53 upregulation, PI3K/AKT downregulation and effective against breast hormone-responsive cancers [128]. PUVA arrest G2/M phase by phospho-Chk1 increment and phospho-Cdc2 decrement in B16F10 murine melanoma cell line [129]. Unfortunately, it is linked toa risk of basal cell, squamous cell, and non-melanoma skin cancers [130,131]. PUVA has been approved by the FD, [132]. PUVA is ideal for superficial applications only, due to low penetration into deep tissues. Therefore, X-PACT has been introduced, which at low X-ray doses photoactivates psoralens and hence mitigates the low penetration problem and adverse effects of PUVA [133].

## 8. Conclusions

Chemotherapy and radiation are the staples for treatment of malignant growth, however, both have serious adverse health symptoms. It is known that tumors have developed many mechanisms at the molecular level enabling cell survival during chemotherapy. Therefore, it is essential to develop novel pharmaceuticals with increased efficacy and reduced toxicity. This review highlights the potential for furanocoumarins to be clinically beneficial in cancer, particularly given their specificity to tumor cells (while sparing normal cells). In vitro investigations have shown that furanocoumarins affect a range of cellular mechanisms, such as apoptosis, autophagy, and cell cycle arrest. ER stress induction mainly caused by NF-κB inactivation, PI3K/Akt inhibition, and p53 modulation. Furanocoumarins are also effective in different MDR cancers that are the main cause of anticancer therapeutics failure. Compounds in this class have also have been shown to positively synergize with commonly used anticancer drugs. The fast absorption of furanocoumarins from food into the human bloodstream is also noteworthy [134]. Furanocoumarins, by inhibiting CYP P450 3A4, not only have anticancer properties but also when co-administered with a low bioavailability anticancer compound can increase oral bioavailability [135]. Thus, as to improve genuine treatments for various sorts of tumors, nanomedicine has developed new strategies coordinated to build the efficacy of medications focusing on tumors and limit their side effects [136,137]. Furanocoumarin-loaded lipid-polymer-hybrid-nanoparticles represent an additional option for sustained release of these molecules to improve efficacy and synergistic effects with other anticancer agents and against MDR cancers [108,138,139]. To date, most focus has been on in vitro studies, making it hard to reach solid conclusions on the efficacy of furanocoumarins in vivo. Nonetheless, studies aimed at characterizing furanocoumarin’s efficacy in vivo as well as clinical studies are encouraging, supporting the need for future studies to better characterize furanocoumarin’s potential as efficacious anticancer treatment modalities.

## Figures and Tables

**Table 1:** Anticancer effects of furanocoumarins in the different reported studies.

Furanocoumarins	Dietary Sources [2,3,4]	In Vitro		In Vivo		Anticancer Mechanisms	References
		Cell Lines	Cytotoxic Concentration	Experimental Model	Dose		
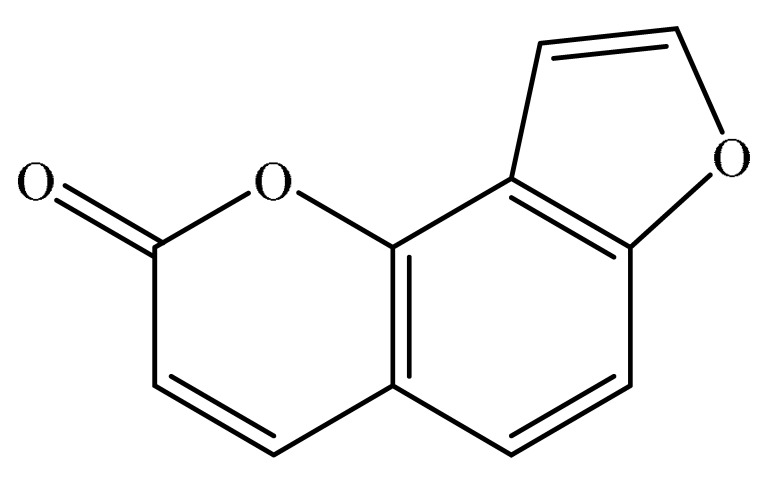 Angelicin	Parsnip	HeLa and SiHa	IC_30_ = 27.8 µM; IC_50_ = 38.2 µM	—	—	Atg3,7 and 12-5 *↑*, G1/G0 arrest	[5]
		A549 (NSCLC)	IC_50_~ 50 µmol	—	—	NF-κB inactivation G2/M phase arrest, cyclinB1 *↓*, cyclin E1 *↓,* and Cdc2 *↓*; Bcl2 *↓*; caspase 3,9 *↑*, Bax*↑* JNK *↑*, ERK *↑*; MMP2, MMP9 *↓*, E-cadherin *↑*	[6]
		SH-SY5Y	IC_50_ = 49.56 μM	—	—	caspase 3,9 *↑*, Bcl-2 *↓*, Bcl-xL*↓*, and Mcl-1*↓*	[7]
		Caki	IC_50_ = angelicin (50–100 μM) and TRAIL (50 ng/mL)	—	—	caspase 3*↑*, c-FLIP *↓*	[8]
		HepG2 and Huh-7	IC50 = 90 ± 6.565 (HepG2); 60 ± 4.256 μM (Huh-7)	—	—	PI3K/Akt inhibition, Bcl-2*↓*	[9]
		—	—	Mouse liver xenograft model (BALB/c-nu/nu mice)	50 mg/kg (16 days)	cancer cell growth *↓*; p-VEGFR *↓*	[9]
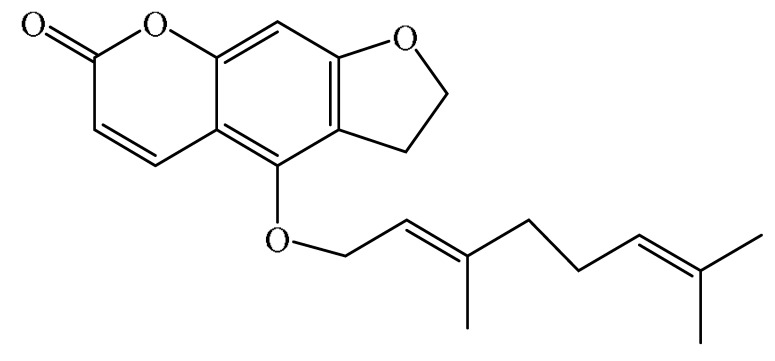 Bergamottin	Carrot, cumin, dill, fig, grapefruit, lemon, lime, orange, parsley, parsnip	A549, H1299	IC_50_ = 50–100 μM	—	—	EMT inhibition, JNK *↑*; PI3K *↓*, Akt*↓*, and mTOR kinases *↓*	[10]
		U87 and U251	IC_50_ = 2–10 μM	—	—	NF-κB inactivation, MMP9 *↓*, STAT3 inhibition, Rac1 inactivation	[11]
		HT-1080	IC_50_ = 5–50 μM	—	—	NF-κB inactivation; MMP2, MMP9*↓*	[12]
		HT-29 and RKO	IC_50_ = 12.5 µM	—	—	caspase-3,8,9*↑*; PARP *↑*; G2/M phase arrest, cyclinA *↓*, cyclin B1*↓*, and Cdc2 *↓*	[13]
		A549 (NSCLC)	IC_50_ = 50 μM	—	—	G2/M phase arrest, cyclinA *↓*, cyclin B1*↓*, and Cdc2 *↓*	[14]
		—	—	Mouse lung xenograft model (BALB/c nude mice)	100 mg/kg (18 days)	↓ cancer cell growth	[14]
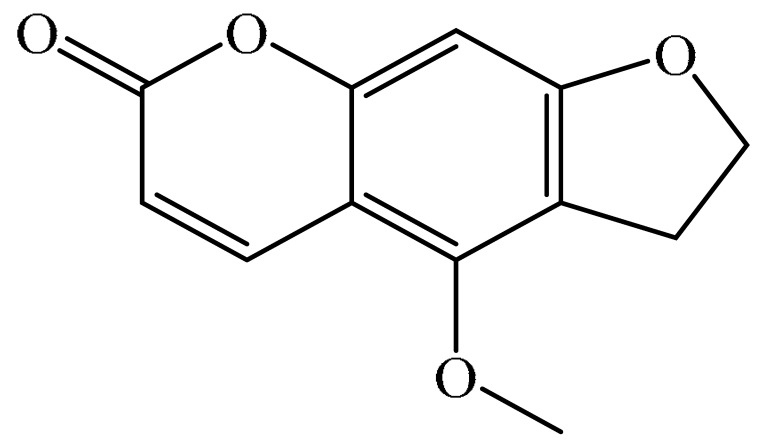 Bergapten or 5-Methoxypsoralen (5-MOP)	Anise, carrot, caraway, celeriac, celery, coriander, cumin, dill, fig, grapefruit, lemon, lime, orange, parsley, parsnip, turnip	DLD-1 and LoVo	IC_50_ = 30 and 50 μM	—	—	G2/M phase arrest, cyclin E *↓*, Cdk2 inhibition; AKT inhibition; p53 *↑*, p21*↑*, PTEN*↑*	[15]
		MCF-7	IC_50_ = 50 μM	—	—	G1-phase phase arrest, P53 *↑*; AKT inhibition	[16]
		Hep-G2	IC_50_ = 25–50 mM	—	—	G2/M phase arrest, Cdk1 inhibition	[17]
			IC_50_ = 25–100 μM	—	—	G2-M phase arrest, P53 *↑* and P21 *↑*	[18]
		A549 (NSCLC)	79.1 ± 2.8% (50 μM)	—	—	G1-phase arrest, P53 *↑*	[19]
		NCI-H460	74.5 ± 3.1% (50 μM)	—	—		
		MCF7 and ZR-75	50 μM	—	—	PTEN *↑*, Beclin1 *↑*, PI3KIII *↑*, UVRAG *↑*, AMBRA, LC3-I to LC3-II	[20]
		HepG2	50 mM	—	—	PI3K/AKT inhibition, LXR (α and β)*↑*	[21]
		—	—	NDEA induced liver cancer (Wistar rats)	25 and 50 mg/kg (14 days)	↓ cancer cell growth	
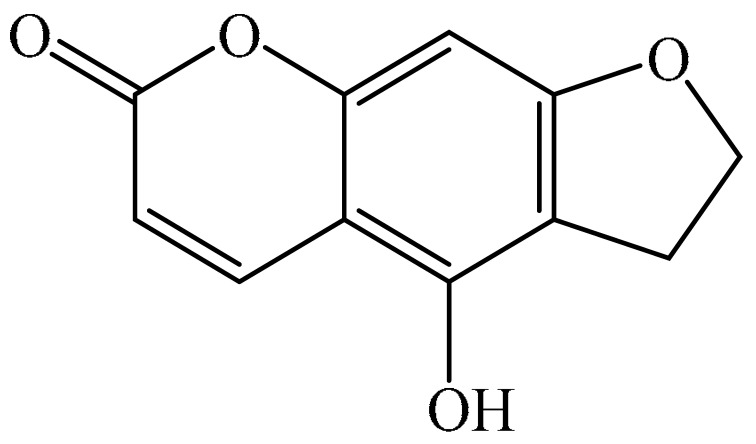 Bergaptol	Carrot, grapefruit, lemon, lime, parsley, parsnip	MCF-7	IC_50_ = 52.2 µM	—	—	G1-phase arrest; caspase 3,9 *↑*, Bax*↑*, Bcl2 *↓*,MMP2 *↓*, MMP9 *↓*, cyt c release *↑*	[22]
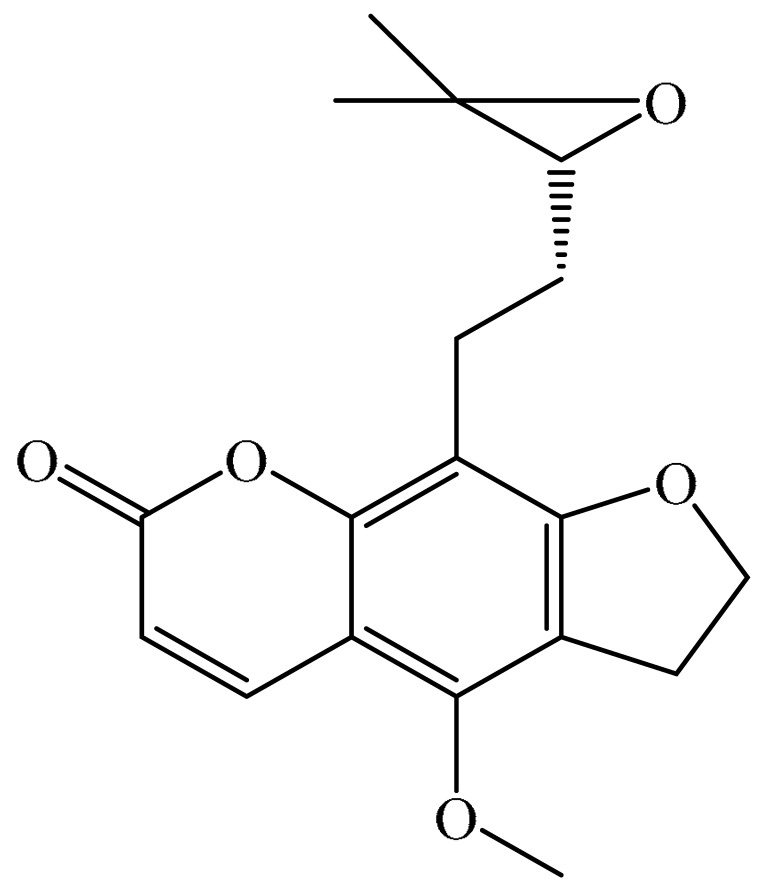 Byakangelicol	Lemon	HCT-15	IC_50_ = 18.1 ± 0.3 μg·mL^−1^	—	—	↓ cell viability	[23]
		A549 (NSCLC)	IC_50_ =14.3 ± 0.2 μg·mL^−1^	—	—		
		SK-OV-3	IC_50_ = 20.2 ± 0.3 μg·mL^−1^	—	—		
		SK-MEL-2	IC_50_ = 21.2 ± 0.3 μg·mL^−1^	—	—		
		XF498	IC_50_ = 28.4 ± 0.3 μg·mL^−1^	—	—		
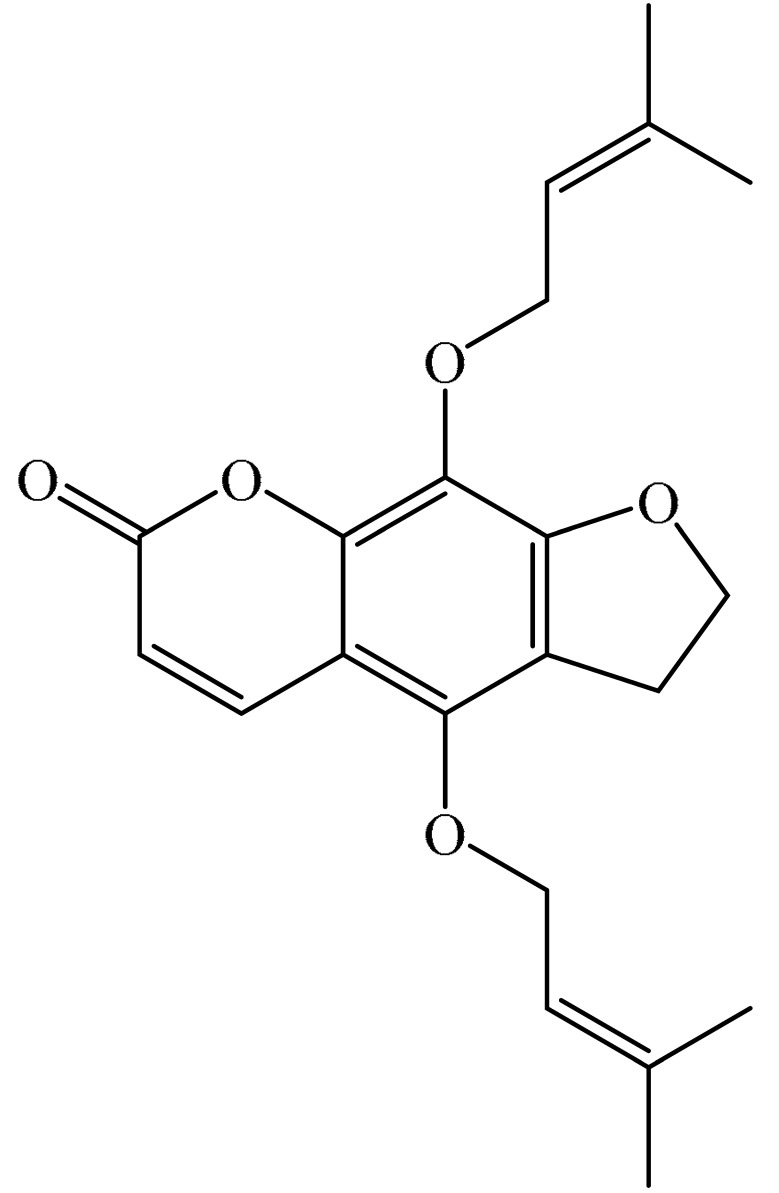 Cnidicin	Lemon	HCT-15	IC_50_ = 7.0 ± 0.2 μg·mL^−1^	—	—	↓ cell viability	[23]
		A549 (NSCLC)	IC_50_ = 6.8 ± 0.1 μg·mL^−1^	—	—		
		SK-OV-3	IC_50_ = 8.8 ± 0.2 μg·mL^−1^	—	—		
		SK-MEL-2	IC_50_ = 8.8 ± 0.2 μg·mL^−1^	—	—		
		XF498	IC_50_ = 7.2 ± 0.3 μg·mL^−1^	—	—		
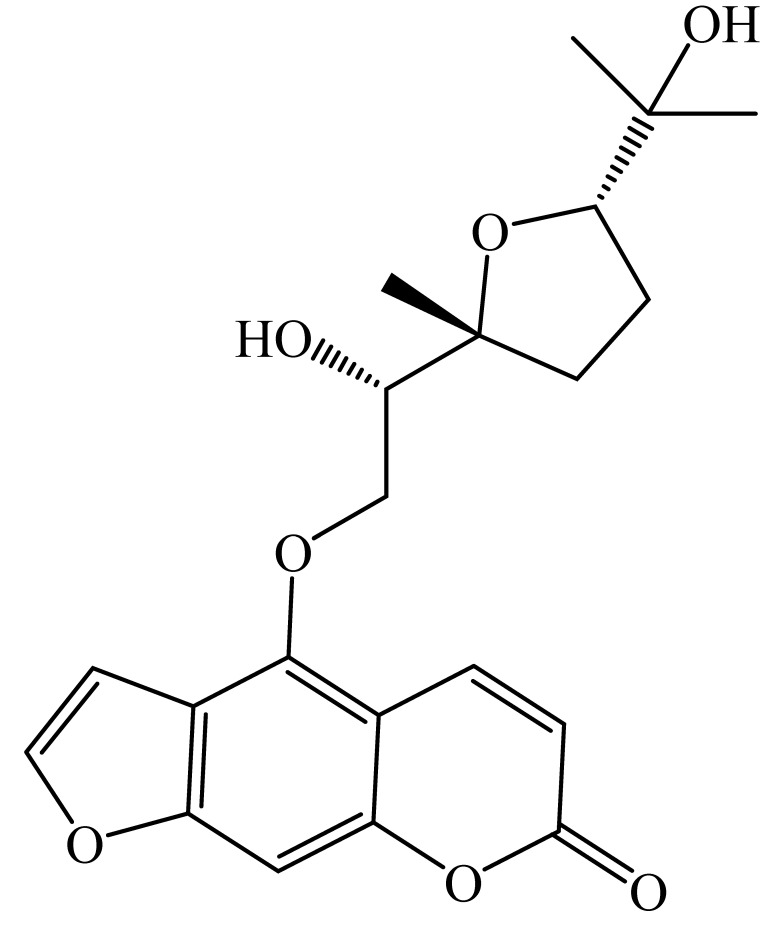 Feroniellin A	*Feroniella lucida* roots	A549	0.25 mM	—	—	NF-κB inactivation Atg5 *↑*, Beclin1 *↑*, mTOR inactivation LC3-I to LC3-II	[24]
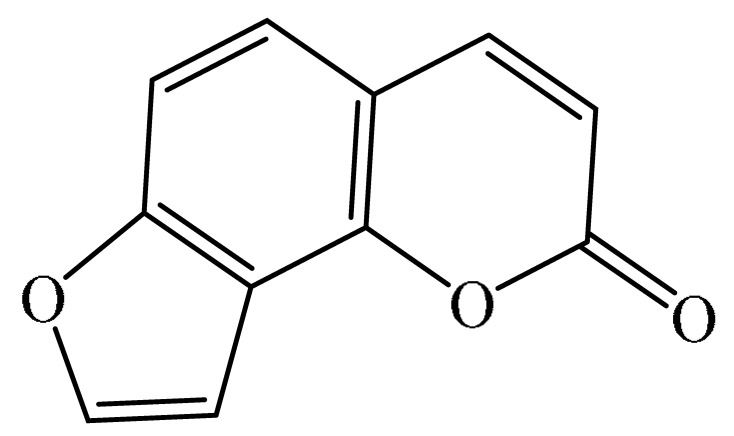 Furanocoumarin A	Fructus liquidambaris	A549	IC_50_ = 65.28 ± 5.36 μM	—	—	P53 *↑*,Bax*↑*, Bcl2 *↓*, caspase 3 *↑*	[25]
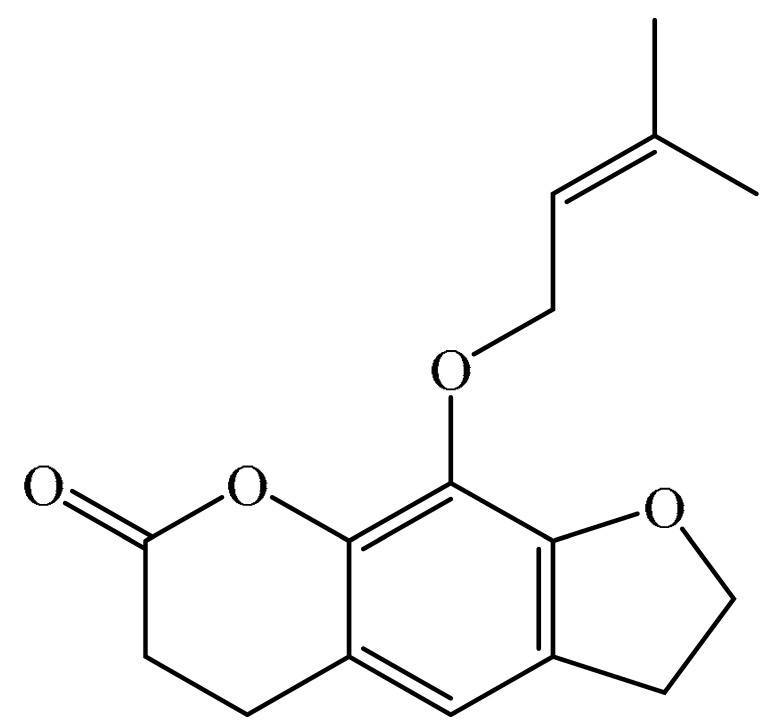 Imperatorin	Lime, parsley	SGC-7901	IC_50_ = 62.6 μM	—	—	promoting G1-phase arrest; PI3K/Akt/m-TOR signaling *↓*	[26]
		HT-29	IC_50_ = 78 µM	—	—	G1-phase arrest; P53 *↑* and P21 *↑*; caspase 3,7 *↑*; Bcl2 *↓*	[27]
		HL-60	10 μM	—	—	caspase 3,9 *↑*; cyt c release *↑*; Bcl2 *↓*, PARP cleavage	[28]
		H23	10 μg/mL	—	—	P53 *↑*,Bax*↑*, Mcl-1*↓*	[29]
		HeLa	200 μM for imperatorin and 5 μM for cisplatin	—	—	Hsp27 and Hsp72 *↓*,LC3 cleavage	[30]
		HCT116	150 μM			mTOR*↓*, p70S6K *↓*, 4E-BP1*↓*, MAPK*↓*, HIF-1*α* inhibition	[31]
		HCT-15	IC_50_ = 19.4 ± 0.3 μg·mL^−1^	—	—	↓ cell viability	[23]
		A549	IC_50_ = 16.4 ± 0.3 μg·mL^−1^	—	—		
		SK-OV-3	IC_50_ =13.7 ± 0.3 μg·mL^−1^	—	—		
		SK-MEL-2	IC_50_ = 14.5 ± 0.2 μg·mL^−1^	—	—		
		XF498	IC_50_ = 12.3 ± 0.5 μg·mL^−1^	—	—		
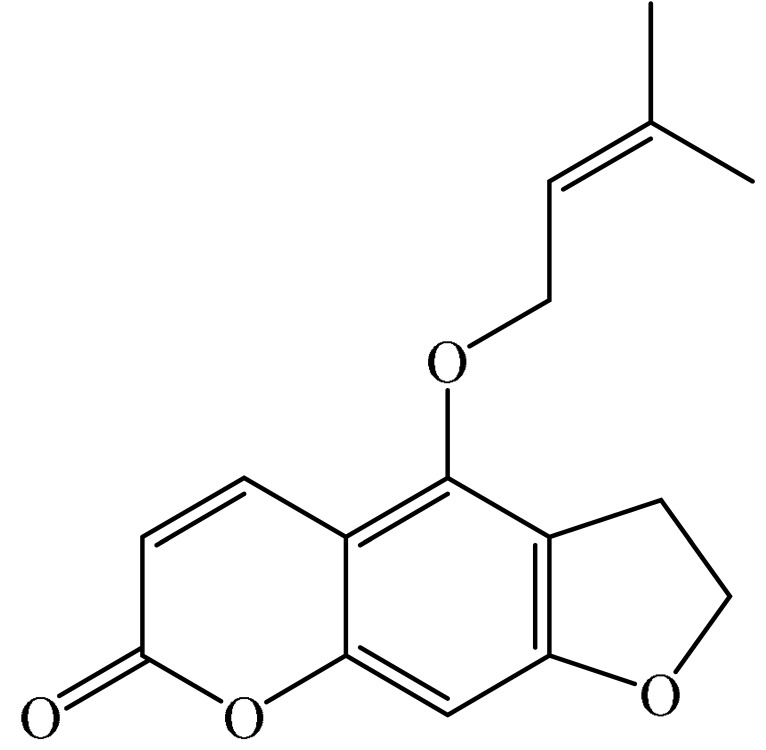 Isoimperatorin	Lime, parsley	SGC-7901	IC_50_ = 18.75 μg·mL^−1^	SGC-7901 cell-induced xenograft model (mice)	10 mg/kg (20 days)	G1-phase arrest; caspase 3,9 *↑*; Bax*↑*, Bcl2 *↓, *Survivin*↓*	[32]
		DU145	100 µM	—	—	G1-phase arrest	[33]
		HCT-15	IC_50_ = 5.6 ± 0.3 μg·mL^−1^	—	—	↓ cell viability	[23]
		A549 (NSCLC)	IC_50_ = 12.2 ± 0.4 μg·mL^−1^	—	—		
		SK-OV-3	IC_50_ = 6.8 ± 0.3 μg·mL^−1^	—	—		
		SK-MEL-2	IC_50_ = 9.9 ± 0.2 μg·mL^−1^	—	—		
		XF498	IC_50_ = 10.7 ± 0.3 μg·mL^−1^	—	—		
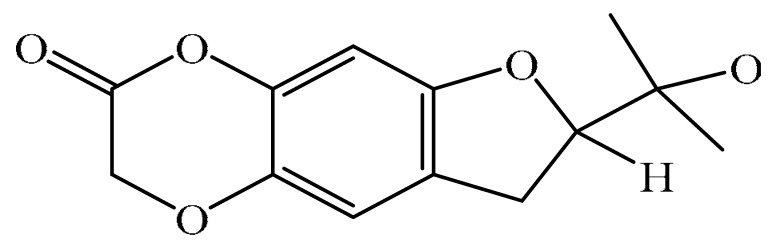 Marmesin	Broad bean, blackberry, raspberry	U937	IC_50_ = 40 µM	Mice	30mg/kg (30 days)	Bax*↑*, Bcl2 *↓*, Bax/Bcl-2 ratio *↑*; G2/M-phase arrest	[34]
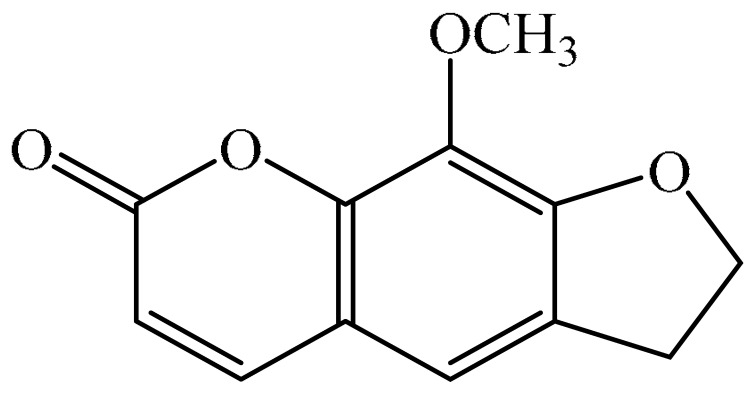 Methoxsalen/Xanthotoxin (8-MOP)	Anise, carrot, caraway celeriac, celery, cumin, dill, grapefruit, lemon, lime, parsley, parsnip, turnip	HepG2	100 µM	—	—	Bax/Bcl-2 ratio *↑,* MMP2 *↓*, MMP9 *↓*, ERK1/2 pathway inhibition, cyt c release *↑*, AIF transposition *↑*	[35]
		SK-N-AS and SW620	IC_50_ = 56.3 µM for SK-N-AS and 88.5 µM for SW620	—	—	Caspase-3,8,9*↑*PI3K/AKT pathway *↓*, Bcl2 *↓*, Bax/Bcl-2 ratio *↑*	[36]
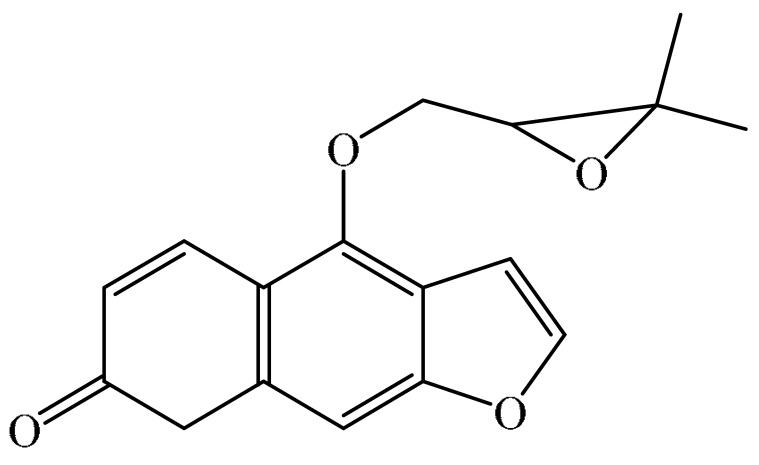 Oxypeucedanin	Lime, parsley	L5178Y (mouse T-cell lymphoma cells)	IC_50_ = 40.33 ± 0.63 µM	—	—	caspase 3,9 *↑*; cyt c release *↑*; Bcl2 *↓*	[37]
		HCT-15	IC_50_ = 3.4 ± 0.3 μg·mL^−1^	—	—	↓ cell viability	[23]
		A549 (NSCLC)	IC_50_ = 9.5 ± 0.3 μg·mL^−1^	—	—		
		SK-OV-3	IC_50_ = 19.3 ± 0.3 μg·mL^−1^	—	—		
		SK-MEL-2	IC_50_ = 16.5 ± 0.2 μg·mL^−1^	—	—		
		XF498	IC_50_ = 16.1 ± 0.5 μg·mL^−1^	—	—		
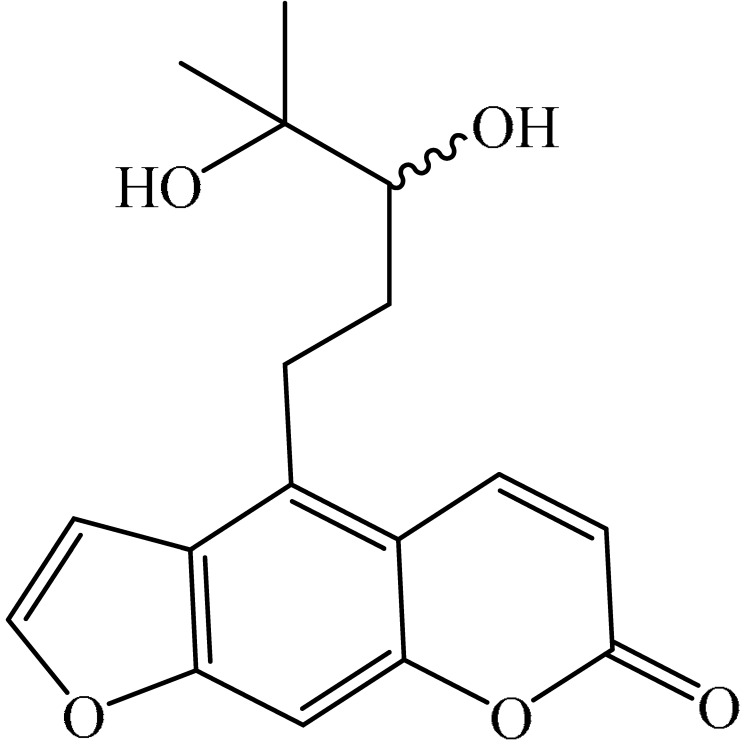 (+)-oxypeucedanin hydrate	*Angelica dahurica* roots	A549, HCT-15, SK-MEL-2, SK-OV-3, XF498	IC_50_ > 30 μg·mL^−1^	—	—		
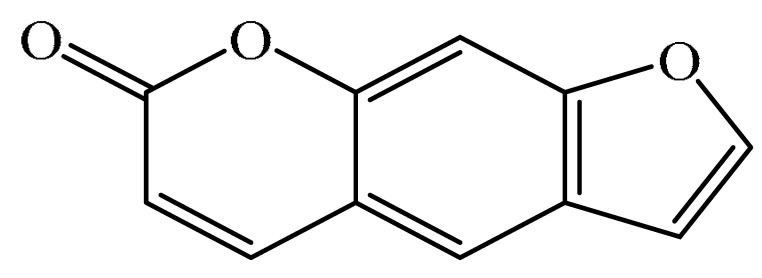 Psoralen	Carrot, celeriac, celery, cilantro, cumin, dill, fig, grapefruit, lemon, lime, parsley, parsnip	KBv200	75.3% (80 μg·mL^−1^)	—	—	NF-κB inactivation G1/S phase arrest, c-FLIP, and IAP inhibition	[38]
		K562	92.4% (80 μg·mL^−1^)	—	—		
		MCF-7	17.32 ± 4.28% (8 μg·mL^−1^)	—	—	G1/G0 phase arrest	[39]
		SMMC7721	40 μM	—	—	G1 phase arrest, cyclin E*↓*, Bax*↑*, Bcl2 *↓*, ER stress, CHOP induction, GADD34*↑*, ATF4*↑*, GRP78*↑*, GRP94	[40]
		MCF-7/ADR	IC_10_ = 8 μg/mL			P-gp efflux function inhibition	[41]
			IC_50_ = 25.59 ± 1.74 µg/mL			P-gp ATPase activity inhibition	[42]
			43.0 µM			EMT inhibition	
			107.5 µM			G0/G1 phase arrest	
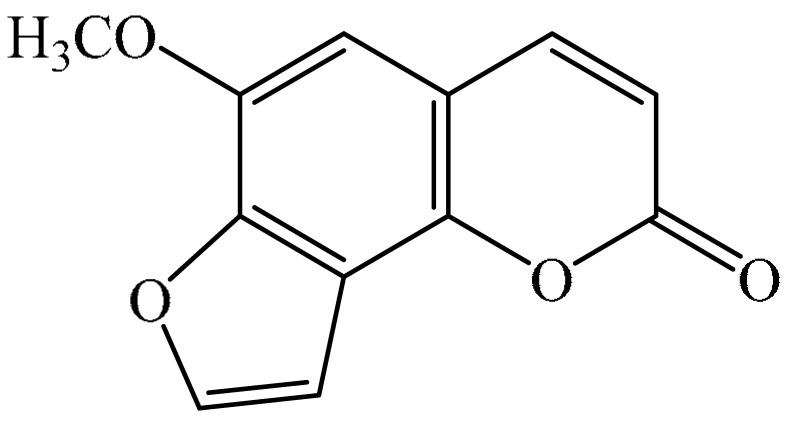 Sphondin	Parsnip	A549	50 μM	—	—	NF-κB inactivation	[43]
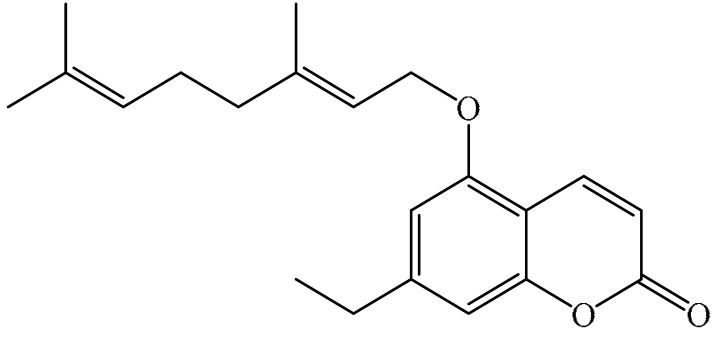 5-geranyloxy-7-methoxy-coumarin	Lime	SW480	25 µM (67%)	—	—	caspase-3,8*↑*; Bcl2 *↓*; p38 MAPK phosphorylation inhibition; G1/G0 phase arrest	[44]

Human cancer cell lines: A549 (NSCLC), NCI-H460 (NSCLC), H23, and H1299 = lung cancer; Caki = renal carcinoma; DLD-1, LoVo, HCT-15, HT-29, HCT116, RKO, SW480 and SW620 = colorectal cancer; DU145 = prostate cancer; HL-60 = promyelocytic leukemia; HeLa and SiHa = cervical cancer; HepG2, Huh-7 and SMMC7721 = liver carcinoma; HT-1080 = fibrosarcoma; KBM-5, K562 = human chronic myeloid leukaemia; KBv200 = oral squamous carcinoma; MCF7 SKBR-3 and ZR-75 = breast cancer; SH-SY5Y and SK-N-AS = neuroblastoma; SK-MEL-2 = melanoma; SK-OV-3 = ovarian cancer; SGC-7901 = gastric cancer cell; U937 = leukemia; U87 and U251 = glioma cells; XF498 = CNS solid tumor; MCF-7/ADR = doxorubicin resistant derivatives of MCF-7 cells overexpressing P-gp; *↑* = increase; *↓* = decrease.
